# Accuracy and Precision of Wearable Devices for Real-Time Monitoring of Swimming Athletes

**DOI:** 10.3390/s22134726

**Published:** 2022-06-23

**Authors:** Gloria Cosoli, Luca Antognoli, Valentina Veroli, Lorenzo Scalise

**Affiliations:** Department of Industrial Engineering and Mathematical Sciences, Università Politecnica delle Marche, 60131 Ancona, Italy; l.antognoli@pm.univpm.it (L.A.); valentina.veroli@gmail.com (V.V.)

**Keywords:** wearable devices, measurement accuracy, measurement precision, swimming, physiological parameters

## Abstract

Nowadays, the use of wearable devices is spreading in different fields of application, such as healthcare, digital health, and sports monitoring. In sport applications, the present trend is to continuously monitor the athletes’ physiological parameters during training or competitions to maximize performance and support coaches. This paper aims to evaluate the performances in heart rate assessment, in terms of accuracy and precision, of both wrist-worn and chest-strap commercial devices used during swimming activity, considering a test population of 10 expert swimmers. Three devices were employed: Polar H10 cardiac belt, Polar Vantage V2, and Garmin Venu Sq smartwatches. The former was used as a reference device to validate the data measured by the two smartwatches. Tests were performed both in dry and wet conditions, considering walking/running on a treadmill and different swimming styles in water, respectively. The measurement accuracy and precision were evaluated through standard methods, i.e., Bland–Altman plot, analysis of deviations, and Pearson’s correlation coefficient. Results show that both precision and accuracy worsen during swimming activity (with an absolute increase of the measurement deviation in the range of 13–56 bpm for mean value and 49–52 bpm for standard deviation), proving how water and arms movement act as relevant interference inputs. Moreover, it was found that wearable performance decreases when activity intensity increases, highlighting the need for specific research for wearable applications in water, with a particular focus on swimming-related sports activities.

## 1. Introduction

In the last few decades, wearable devices (smartwatches, in particular) have spread in several application fields, from healthcare (e.g., symptoms detection and tracking in COVID-19 pandemics [1], monitoring of cardiac-related diseases [2], and so on) to sports (for example, climbing [3], football [4], and swimming [5]), through supporting elderly people [6] and well-being assessments [7]. The use of wearable devices is often combined with Artificial Intelligence (AI) technologies, used for prediction or classification purposes. For example, in the literature there are studies aimed at the classification of emotions [8], detection of stimuli [9], and rehabilitation purposes [10]. In particular, in sports applications, wearables are employed to monitor aspects such as training efficiency [11] and athletes’ performances [12].

In recent years, the use of wearables has rapidly increased, thanks to their multiple advantages, such as usability, low cost, the capability of acquiring different vital signs, and availability in many different segments, hence satisfying different users’ needs. Given their massive usage, it is important to pay proper attention to the measurement accuracy and precision of these devices, particularly since, in the near future, they could play a pivotal role in the telemedicine and eHealth sectors [13].

In sports-related applications, particular care should be given to movement artefacts since signal acquisition during activities is surely more challenging, particularly when photoplethysmographic (PPG) sensors are employed. In fact, PPG sensors are prone to movement artefacts, as well as subject to variations due to physiological variability of subjects (e.g., skin tone [14], perfusion level [15], skin temperature [16]), and to setup parameters (such as wavelength [17] and led configuration [18]). Different strategies to minimize the deteriorating effects of motion artefacts are reported in the literature. For example, some LED colours have been proved to be more sensitive to motion artefacts, such as green light, being less penetrating [19], but for this reason providing a better Signal-to-Noise Ratio (SNR) [20], also with different skin tones [17]. There are also sensors (e.g., MAX86141 by Maxim Integrated) using an accelerometer to detect movement and adopt a redundant double optical path so that the outputs are correlated to enhance the measurement accuracy of the cardiac frequency. Other devices, such as Empatica E4, do not provide data from the PPG when movement artefacts are detected through an accelerometer. Therefore, raw data from the PPG sensor can show some gaps that must be corrected by proper algorithms [21].

Among the plethora of possible sports activities, the authors have focused their attention on swimming, one of the most complex and complete options. This is not only for the involved movements but also for the water environment, whose effect should be taken into account when performing any type of measurement (e.g., heart rate (HR) [22], oxygen consumption (VO_2_) [23], carbon dioxide production (VCO_2_) [24], and so on). Indeed, for PPG-based sensors, the PPG–skin interface is fundamental. For example, Scardulla et al. proved that the contact pressure plays a pivotal role in the determination of the signal quality [25]. Moreover, water interrupts the direct contact between PPG sensors and the skin, hence hindering the optical signal path. Considering movement artefacts, it is worth mentioning that the arms rhythmic movement in swimming activities influence the quality of the collected data. For these reasons, carrying out measurements with PPG sensors in water is very challenging. On the other hand, PPG-based sensors are the most diffused wearable technology, particularly where smartwatches are concerned. Chest-worn devices are generally more accurate since the sensor–skin contact is better and the thoracic area is less subjected to movement artefacts; sometimes cardiac belts also have electrodes, which are able to gather the electrical signal related to cardiac activity.

In the literature there are several different types of studies which employ wearable devices for physiological monitoring during swimming activities. For example, Olstad and Zinner considered the optical sensor OH1 positioned underneath the swim cap at the temple, together with a Polar H10 worn underneath the swimsuit, besides a smartwatch (Polar M600) [26]. They found an excellent agreement between the chest-strap and OH1 sensor, whereas worse results were obtained for the smartwatch. Furthermore, it is worth underlining that the sensor on the temple allows the minimization of the water constriction effect on vessels and help to avoid the wrist reduced peripheral resistance; movement artefacts are significantly lowered.

Additionally, the recording of ECG signals underwater is challenging because common electrodes can lose skin contact; particular electrodes for wet environments were proposed. For example, Ji et al. employed a stretchable metal-polymer composite film substrate with a dopamine-containing polymer coating, which showed a longer resistance before detachment with respect to standard electrodes [27].

Regardless of the type of acquisition sensor, in order to properly interpret the gathered data, the metrological characterisation of wearable devices is fundamental. Measurement results should be always considered with the measurement accuracy and precision of the employed instrumentation (since the same quantities, e.g., HR, measured with different sensors can be affected by diverse uncertainty values [28]). For these reasons, it is strongly advisable to validate wearable devices against standard apparatuses (e.g., an electrocardiograph for cardiac-related parameters), considering the same operating environment where the measurements will be performed. However, no test protocols have been standardised so far for the validation of innovative measurement devices in comparison to reference instrumentation. Hence, the data available in the literature are quite inhomogeneous and can be scarcely compared [29]. Furthermore, measurement accuracy and precision of commercial devices are seldom available and scarcely investigated in the literature, and manufacturers rarely provide technical specifications related to measurement uncertainty; even when data on accuracy/precision are given, the test protocol adopted to obtain them is not available.

This study aimed to compare the metrological performance (in terms of measurement accuracy and precision) of wearable devices declared suitable for in-water use and to evaluate the effect of both water and activity on the obtained metrics. In particular, the authors would like to propose a test procedure focused on swimming activities, aiming at the metrological characterisation of wearable devices, compared to a reference instrument, in the context of heart rate assessment. Hence, the main contribution is the proposal of a test protocol specific for wearable devices to be applied in water. In addition, the test protocol should be adaptable to different contexts and also to the analysis of different vital parameters (e.g., energy expenditure or performed activity). Furthermore, this work provides metrological performance data that, to the best of the authors’ knowledge, are currently not available in the literature.

The rest of the paper is organized as follows: Section 2 describes the study methodology and the used sensors and data processing techniques; Section 3 reports the results; in Section 4, the Authors provide their comments on this study findings and propose some future perspectives.

## 2. Materials and Methods

In this study, the test population consisted of 10 healthy subjects, aged 17 ± 3 years, with a BMI of 19.80 ± 1.21 kg/m^2^. The main characteristics of the participants are reported in Table 1. The subjects were asked not to smoke or take medications in the few hours preceding the experiment. They voluntarily took part in the experimental campaign. The study objectives and methodology were clearly explained before the test execution, and the measurements were performed in compliance to the WMA Declaration of Helsinki [30]. All the subjects (or a parent, in case of minors) signed an informed consent before the tests.

**Figure 1:**
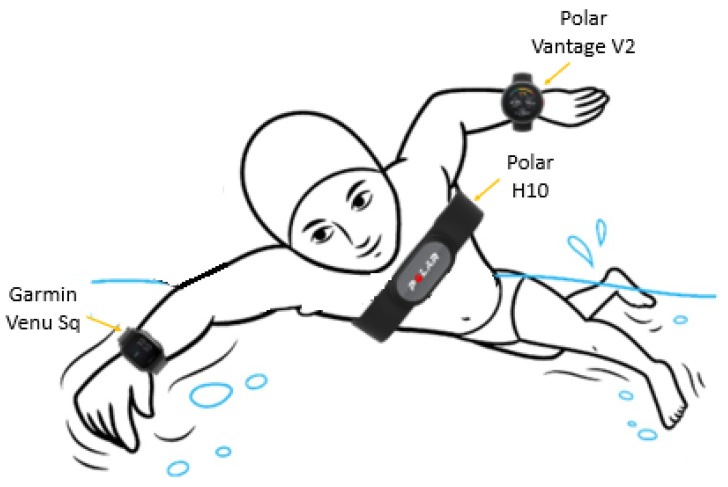
Device placement for in-water test: the two smartwatches are worn on the swimmer’s wrists (Polar Vantage V2 on the left wrist, Garmin Venu Sq on the right one), whereas the Polar H10 cardiac belt is on the thorax. The same devices were used for laboratory test acquisitions.

Physiological data were acquired by means of three wearable devices, namely a cardiac belt (used as reference instrument) and two smartwatches (Figure 1):

- Polar H10: A chest-strap, cardiac belt device embedding high-quality electrodes; it can be easily maintained in position thanks to silicon dots and an improved buckle. It has been considered the reference device for HR measurement; in fact, chest-worn wearable devices are generally more accurate with respect to wrist-worn ones [31], mainly for the different sensing principle, which is based on electrodes (hence, an electrical signal is acquired) instead of PPG (optical signal) and also due to the fact that they are placed on the thoracic area, in correspondence to the cardiac muscle [29]. Moreover, it is commonly used as a gold standard in the literature [32,33];
- Polar Vantage V2 smartwatch [34]: A lightweight smartwatch suitable for sports and fitness activities. Its battery life is 40 h in training modality and up to 7 days in sport-watch mode (sampling of 1 Hz for recording HR–the cardiac-related signal has to be sampled at a higher frequency to avoid aliasing issues). It is based on a 10-LED PPG sensor;
- Garmin Venu Sq [35]: A widespread smartwatch, suitable for sports activities, able to derive a plethora of parameters, both directly (e.g., HR) and indirectly (e.g., respiratory rate). The measured results (in terms of HR series) are provided with a frequency of 1 Hz (but the optical signal is clearly sampled at higher rate), 24 h a day, 7 days a week.

The technical specifications of the used wearables are reported in Table 2. It is worth noting that a devices measurement accuracy and precision are not provided by manufacturers. Few studies in the literature report data related to the metrological characterisation of the employed devices. For example, Gilgen-Ammann et al. found a signal quality (i.e., relative number of correctly detected RR intervals) of 99.6% for the Polar H10 cardiac belt [36], which is often considered a reference instrument for HR measurement [33,37]). Polar manufacturer published a white paper in 2019, reporting an error of HR < 4% in all the tested activities (i.e., running, cycling, weight training, and a combination of them), proving that its performance in measuring RR intervals is excellent [38]. Nuuttila et al. [39] tested Polar Vantage V2 for HR and HRV measurements in rest conditions, and found a very good agreement with reference (correlation coefficient > 0.99) and a slight overestimation of the log-transformed root mean square of successive RR intervals–lnRMSSD–(mean bias of 0.20 ms and 0.17 ms in laboratory and nocturnal recordings, respectively). They concluded that the smartwatch is suitable for recreational athletes. To the best of the authors’ knowledge, at present, there are no studies reporting the metrological performance of the Garmin Venu Sq. It is worth underlining that, being that these devices are relatively new in the market, at present, very few studies have employed them, especially with metrological characterisation purposes.

Of note, all the employed devices are water-resistant, even if water can cause issues to the general measurement process, especially when based on a PPG sensor. Moreover, some technical specifications related to specific devices should be considered. For example, Polar H10 can be used during water activities, but the data can be accessed afterwards through the app and not in real-time, since Bluetooth wireless technology does not work in water. Moreover, the presence of water could disturb the electrical signals, acting as a conductive path and preventing the cardiac activity detection through the heart rate sensor; hence, guaranteeing a suitable and stable contact with the skin is fundamental (e.g., wearing the cardiac belt under the bathing suit, maintaining the sensor in position). Additionally, Polar Vantage V2 smartwatch is suitable for heart rate monitoring during swimming tasks (water resistance up to 100 m, adapt for frequent use in water but not for scuba diving), but the results could be non-optimal. Polar devices are water-resistant in compliance with the international standards ISO22810 and IEC60529. Garmin Venu Sq is water-resistant up to 5 atm, hence it is suitable for swimming activities.

**Figure 2:**
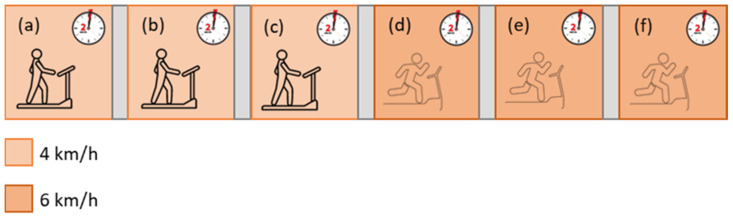
Laboratory test protocol (after initial recording at rest): exercises on treadmill, at two different velocities, namely 4 km/h (**a**–**c**) and 6 km/h (**d**–**f**).

The selected wearable devices were simultaneously worn by each of the tested subjects to acquire physiological data. The experimental tests were conducted both in dry and wet (in the swimming pool) conditions to compare the metrological performances of the tested devices in the two conditions, thus evaluating the (degrading) effect of water on wearable sensors. In the dry case (Figure 2), a treadmill was employed, and two different velocities were selected to obtain two activity intensities. Each subject executed three repeated 2-min tests at 4 km/h and three repeated tests at 6 km/h (slope of zero). Additionally, an initial 5-min acquisition in resting conditions was recorded.

**Figure 3:**
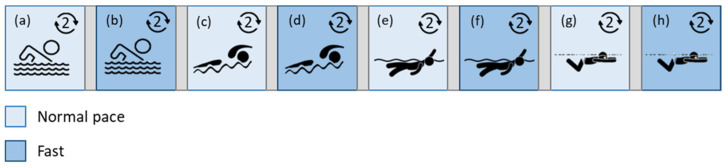
In-water test protocol (after initial recordings at rest): free-style (**a**,**b**), butterfly (**c**,**d**), backstroke (**e**,**f**), and breaststroke (**g**,**h**) laps, at two diverse paces (normal and fast).

Regarding the in-water setup (Figure 3), at first, a 5-min acquisition was performed in dry conditions before entering the water. Then, another 5-min acquisition was carried out in the swimming pool to have traces acquired both in wet and dry conditions and at rest (to discriminate between the effects of water and activity). After the two recordings on the subject at rest, four swimming activities (different styles, involving different movements, possibly affecting the results in a different way) at diverse intensities were monitored, in particular:

- 4 laps free style;
- 4 laps butterfly stroke;
- 4 laps backstroke;
- 4 laps breaststroke.

For each swimming stroke, 4 laps were performed with different intensities: the first two at a normal pace, the second two swimming fast. Clearly, the perceived intensity is subjective, but this was done to experiment two different conditions, more challenging and less challenging, to verify the wearables’ performance depending on the different motion intensities. In fact, it is generally known that performance decreases while intensity grows, since the sensor–skin contact deteriorates and the signal quality decreases [40].

Data were processed in MATLAB environment. At first, data were pre-processed; in particular, data were visually inspected to identify any major issues in the acquisition. Then, data from the different wearables were synchronized thanks to the timestamps provided by the apparatuses. Hence, measurement accuracy and precision were evaluated through standard methods, namely:

- Analysis of deviations: at first, deviations were computed as differences between HR series measured through each smartwatch and cardiac belt (reference instrument). Then, their distribution was evaluated, and the mean and standard deviation values of the obtained deltas were computed, being related to the accuracy and precision of the measurement. More in detail, a coverage factor of 2 (k = 2) was chosen to express the statistical confidence of the measurement. In addition, a Bland–Altman plot [41] was derived. This graphical representation consists of plotting the measurement deviations against the expected value, which is obtained as the average between the measurements performed by the tested device (smartwatch) and the reference instrument (cardiac belt). A Bland–Altman plot helps evaluate the agreement between two measurement techniques; in particular, the mean deviation corresponds to the mean value available on the *y*-axis and is consistent with the measurement accuracy. Furthermore, the related confidence interval at 95%, computed as the mean deviation plus/minus the corresponding standard deviation multiplied by a factor equal to 1.96, can be obtained and related to the measurement precision (related to the expanded uncertainty with a coverage factor of 2);
- Correlation analysis: the Pearson’s coefficient (ρ) was computed to assess the linear correlation between the tested device (smartwatch) and the reference one (cardiac belt). The strength of the relationship was considered high when ρ > 0.7, moderate when 0.3 < ρ < 0.7, and low when ρ < 0.3 [42]. Additionally, the interpolating curve was considered to verify the linearity of the relationship.

Finally, the mean absolute percentage error (*MAPE*), as defined in (1), was evaluated, where *HR_test_* is the *HR* measured by the tested device—smartwatch type, whereas *HR_ref_* is the one recorded by the reference instrument.

(1) $$MAPE=\left|\frac{HR_{test}-HR_{ref}}{HR_{ref}}\right|\cdot 100$$

The obtained performances were compared in wet and dry conditions to evaluate the effect of water on the measurement accuracy and precision. Then, also the effect of activity was evaluated, comparing results during resting and moderate activities.

## 3. Results

In this section, the authors report the results in terms of the metrological performance of the tested smartwatches (Polar Vantage V2 and Garmin Venu Sq) in comparison to the chest-strap device (Polar H10) that was used as reference.

**Figure 4:**
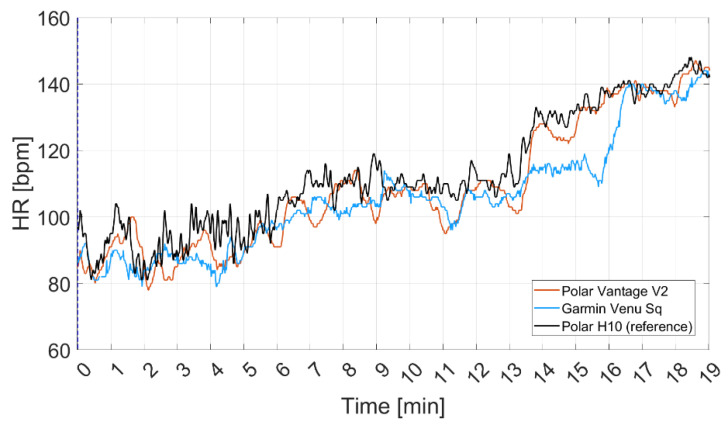
Example of the signals acquired through the two tested smartwatches (Garmin Venu Sq and Polar Vantage V2) and the reference device (Polar H10) in case of dry conditions.

An example of the time series of the signals acquired by the three different sensors is reported in Figure 4 (dry conditions). It is possible to see that agreement between test and reference device is affected by activity (increasing over time, according to the test protocol, see Figure 2).

### 3.1. Evaluation of the Effect of Water: In-Water vs. Dry Acquisitions

The first comparison was performed to evaluate the effect of water. Hence, results related to measurement accuracy and precision are reported in the next subsections in the two test conditions.

#### Measurement Accuracy and Precision

**Figure 5:**
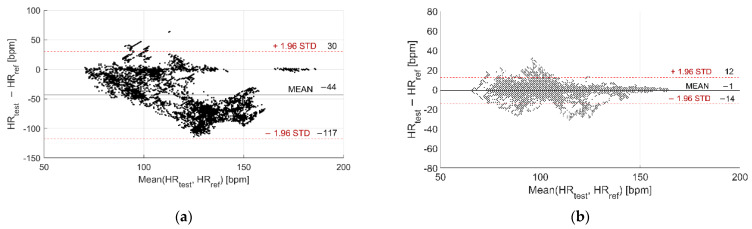
Bland–Altman plot related to (**a**) In-water and (**b**) Dry acquisitions, including recordings both at rest and during moderate physical activities performed with Garmin Venu Sq (reference instrument: Polar H10).

**Figure 6:**
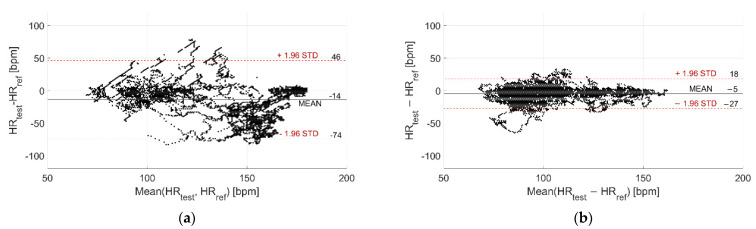
Bland–Altman plot related to (**a**) In-water and (**b**) Dry acquisitions, including recordings both at rest and during moderate physical activities performed with Polar Vantage V2 (reference instrument: Polar H10).

The Bland–Altman plots related to in-water and dry acquisitions are reported in Figure 5 and Figure 6 for Garmin Venu Sq and Polar Vantage V2, respectively. All the recordings were considered together, both during moments of rest and physical activities, in order to evaluate possible variations of metrological performance with activity intensity, for example, through the Bland–Altman plot. It can be noted that precision significantly worsens when tests are performed in water with respect to dry conditions: the CI 95% passes from [−14, 12] bpm to [−117, 30] bpm for Garmin Venu Sq, from [−27, 18] bpm to [−74, 46] bpm for Polar Vantage V2. The same deterioration can be observed for accuracy: the mean deviation increases from −1 bpm to −44 bpm for Garmin Venu Sq, from −5 bpm to −14 bpm for Polar Vantage V2. Moreover, concerning in-water tests, it seems that deviations are negative for high values of HR (>110 bpm), as if both the smartwatches underestimate HR in the upper part of the measurement range. Polar Vantage V2 seems to be more robust against the effects of both water and physical activity (and, hence, movement artifacts), with an average deviation reduced of approximately 5 times; this can be due both to hardware and software factors, impacting on sensor-skin contact and data processing, respectively.

**Figure 7:**
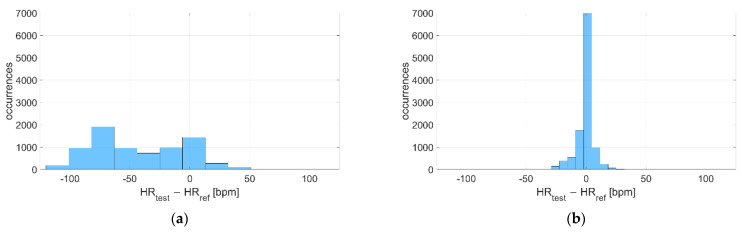
Distribution of deviations related to (**a**) In-water and (**b**) Dry acquisitions, including recordings both at rest and during moderate physical activities performed with Garmin Venu Sq (reference instrument: Polar H10).

**Figure 8:**
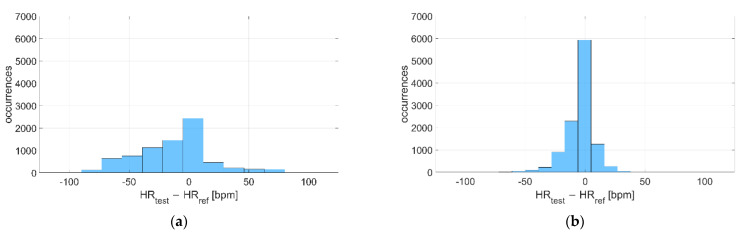
Distribution of deviations related to (**a**) In-water and (**b**) Dry acquisitions, including recordings both at rest and during moderate physical activities performed with Polar Vantage V2 (reference instrument: Polar H10).

The deviations with respect to the reference instrument are reported in Figure 7 and Figure 8 for Garmin Venu Sq and Polar Vantage V2, respectively. It can be noted that the distribution is much narrower when tests are performed in dry conditions, since the standard deviation of the distribution is very lower (7 bpm vs. 37 bpm for Garmin Venu Sq, 12 bpm vs. 30 bpm for Polar Vantage V2). Moreover, for Garmin Venu Sq, the distribution of deviations for in-water recordings seems almost bimodal. Water has a deteriorating effect on the metrological performance of the tested devices, both in terms of precision and accuracy.

**Figure 9:**
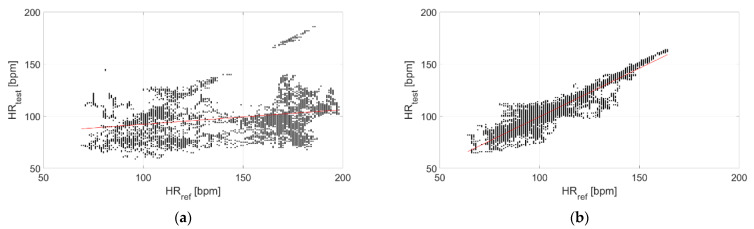
Correlation of measurements performed through Garmin Venu Sq with respect to the reference sensor (Polar H10) related to (**a**) In-water and (**b**) Dry acquisitions, including recordings both at rest and during moderate physical activities.

**Figure 10:**
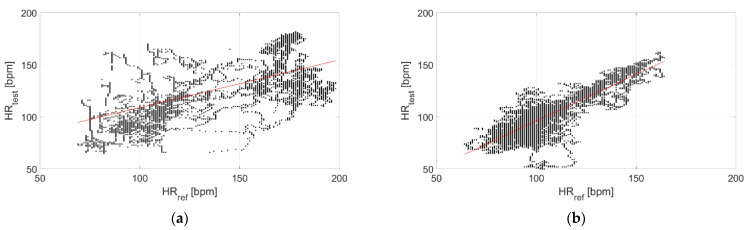
Correlation of measurements performed through Polar Vantage V2 with respect to the reference sensor (Polar H10) related to (**a**) In-water and (**b**) Dry acquisitions, including recordings both at rest and during moderate physical activities.

The correlation with the measurements performed through the reference sensor is reported in Figure 9 and Figure 10 for Garmin Venu Sq and Polar Vantage V2, respectively. It can be observed that correlation is much stronger when tests are performed in dry conditions; in fact, the Pearson’s correlation coefficient diminishes from 0.95 to 0.26 for Garmin Venu Sq and from 0.85 to 0.59 for Polar Vantage V2 when passing from dry to in-water tests. Hence, the correlation with respect to the reference instrument can be considered strong for dry conditions of the test, whereas it is low and moderate for in-water recordings for Garmin Venu Sq and Polar Vantage V2, respectively.

The results are summarized in Table 3; it should be noted that accuracy and precision (with a coverage factor k = 2) are derived from the analysis of the deviations, whereas CI95% is obtained through the Bland–Altman plot. Thus, it results that both precision (quantifiable as 2∙σ or by CI95%) and accuracy (corresponding to the mean deviation) are worse when recordings are made in water. These aspects are well reflected by the MAPE values, which passing from dry to in-water test conditions become significantly higher (from 4.05% to 29.95% in the case of Garmin Venu Sq, from 8.00% to 17.17% for Polar Vantage V2).

If measurement accuracy and precision are evaluated separately at rest and during activity (Table 4), it is possible to observe that accuracy is significantly worsened by the presence of water, as well as precision (both accuracy and confidence interval indicate wider ranges). Moreover, also MAPE increases for in-water tests. However, concerning activity, its effect is not so marked if in-water and dry tests are considered separately. In fact, for dry tests, the Pearson’s correlation coefficient seems better when recordings are made during activity. Instead, for in-water tests, activity degrades all the performance metrics. Consequently, it should be thoroughly considered that a worse performance is to be expected when smartwatches are employed for monitoring HR during in-water activities.

### 3.2. Evaluation of the Effect of Activity: Acquisitions during Resting vs. Activity

The effect of activity, presumably degrading the metrological performance of PPG-based devices mainly due to movement artifacts, was evaluated. As stated above, however, its negative effect seems to be significant only for in-water test conditions. The results related to measurement accuracy and precision are reported in the next subsections in the two test conditions, considering both dry and in-water tests together.

#### Measurement Accuracy and Precision

**Figure 11:**
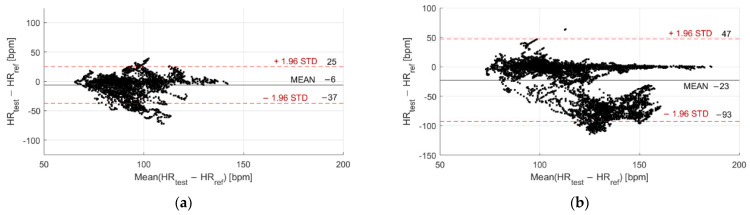
Bland–Altman plot related to recordings performed (**a**) Resting and (**b**) During activities, including recordings both at in-water and dry conditions performed with Garmin Venu Sq (reference instrument: Polar H10).

**Figure 12:**
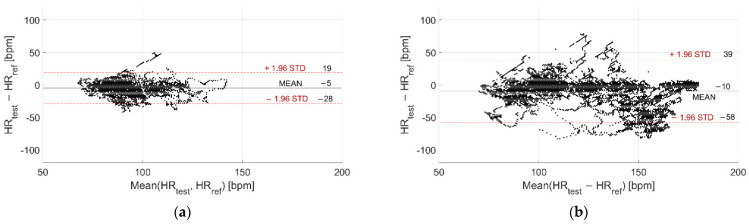
Bland–Altman plot related to recordings performed (**a**) Resting and (**b**) During activities, including recordings both at in-water and dry conditions performed with Polar Vantage V2 (reference instrument: Polar H10.

The Bland–Altman plots related to acquisitions performed at rest and during activities are reported in Figure 11 and Figure 12 for Garmin Venu Sq and Polar Vantage V2, respectively; both dry and in-water conditions were evaluated together. It can be noted that accuracy worsens with activity, even if the movement effect is quite well mitigated in the case of Polar Vantage 2, where the mean deviation passes from −5 bpm at rest to −10 bpm during activities (contrarily to Garmin Venu Sq, where bias is −6 bpm at rest and −23 bpm during activities). Additionally, CI 95% widens, indicating a worse precision during activity. Moreover, as already mentioned before, deviations are negative for high HR values, meaning that smartwatches underestimate HR when the activity level is moderate, implying high HR.

**Figure 13:**
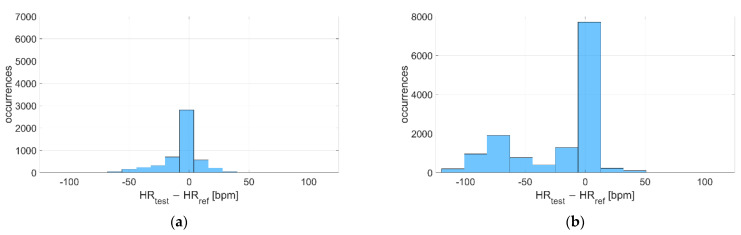
Distribution of deviations related to (**a**) Resting and (**b**) During activities, including recordings both at in-water and dry conditions performed with Garmin Venu Sq (reference instrument: Polar H10).

**Figure 14:**
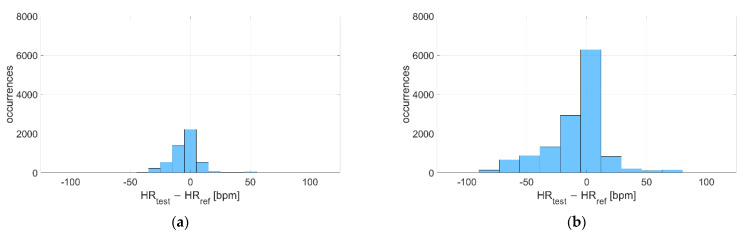
Distribution of deviations related to (**a**) Resting and (**b**) During activities, including recordings both at in-water and dry conditions performed with Polar Vantage V2 (reference instrument: Polar H10).

The deviations with respect to the reference instrument are reported in Figure 13 and Figure 14 for Garmin Venu Sq and Polar Vantage V2, respectively. It can be noted that the distribution of deviations is always Gaussian-like, except for recordings during activity related to Garmin Venu Sq; this was already observed previously for in-water acquisitions, meaning that both water and activities significantly worsen the quality of data, whose deviations, contrarily to what expected, show an almost bi-modal distribution.

**Figure 15:**
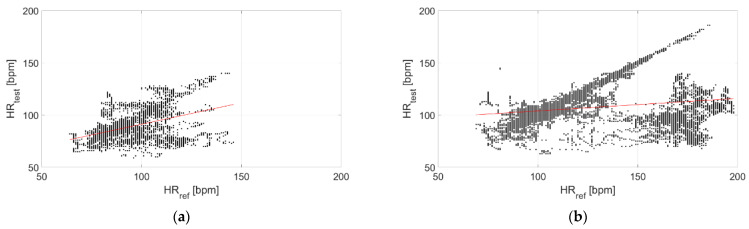
Correlation of measurements performed through Garmin Venu Sq with respect to reference sensor (Polar H10) related to recordings made (**a**) In resting and (**b**) During activities, including recordings both at in-water and dry conditions (reference instrument: Polar H10).

**Figure 16:**
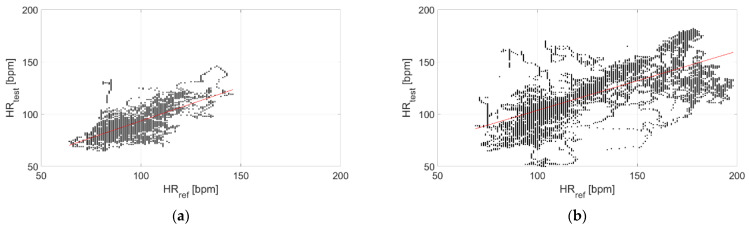
Correlation of measurements performed through Polar Vantage V2 with respect to reference sensor (Polar H10) related to recordings made (**a**) Resting and (**b**) During activities, including recordings both at in-water and dry conditions (reference instrument: Polar H10).

The correlation with the measurements performed through the reference sensor is reported in Figure 15 and Figure 16 for Garmin Venu Sq and Polar Vantage V2, respectively. It can be observed that correlation is better at rest, with activities heavily impairing the quality of the recorded data. Results show moderate correlations, with the exception of low correlation for data recording through Garmin Venu Sq during activities. It is worth underlining that the obtained values of Pearson’s correlation coefficient were influenced by in-water tests for the evaluations reported in this section (otherwise, better results are achieved, see Table 3).

The results are summarized in Table 5. It results that activity impairs both accuracy and precision (when considering both dry and in-water test conditions together), to a greater extent in the case of Garmin Venu Sq with respect to Polar Vantage V2. This is confirmed by a higher MAPE. However, considering both in-water and dry test conditions, it is beyond doubt the negative effect of water on data quality and, hence, on the metrological performance of the tested wearable devices.

Thanks to the results reported in Table 4, where both in-water/dry test conditions and rest/activity are considered separately, it is possible to attribute a greater weight to the influence of water with respect to movements associated to activity execution.

## 4. Discussion and Conclusions

In this study, the authors evaluated the metrological performance of two wrist-worn wearable devices, namely Polar Vantage V2 and Garmin Venu Sq, with respect to a cardiac belt considered as reference (i.e., the Polar H10). The main objective of the study was to evaluate the accuracy and precision of such devices during swimming activities, where their application results are particularly interesting in both training and competition sessions, supporting the coaches in enhancing the athletes’ performance. Since, to the best of the authors’ knowledge, in the literature there is no test protocol available for validating wearable devices in swimming, the authors designed a test protocol including both rest and activities periods (i.e., walking on a treadmill and swimming), to evaluate the effect of movement on the signal quality, as well as possible variations of metrological performance with the HR range. Moreover, both dry and in-water tests were planned to evaluate how water can affect the results since, in PPG-based sensors, it undoubtedly hinders the optimal contact between the skin and the sensor itself. Results show that both precision and accuracy worsen in in-water tests (the measurement deviation increases by 9–43 bpm for mean value and by 37–61 bpm for standard deviation), as well as with increasing activity intensity (with an increase in terms of measurement deviation of 5–17 bpm and 24–39 bpm, respectively for mean and standard deviation). This proves how water and arms movement act as relevant interference inputs for measuring heart rate through PPG-based wearable devices. The measuring principle of smartwatches is prone to movement artefacts, and their performance is heavily influenced by the goodness of the sensor-skin contact. In fact, these disturbance effects are generally mitigated in the case of cardiac belts based on electrodes. Moreover, it can be stated that the metrological performance of Polar Vantage V2 is better with respect to Garmin Venu Sq in terms of accuracy (with comparable precision) if we are interested in swimming applications involving both the presence of water and activity. In particular, the Polar Vantage V2 results show an accuracy of −5 bpm at rest in dry conditions, with a precision of ±19 bpm (coverage factor k = 2); however, the metrics worsen due to the presence of water and movement artefacts, showing an accuracy of −18 bpm with a precision of ±68 bpm for in-water tests during activity. In the case of the Garmin Venu Sq, accuracy and precision are equal to −1 bpm and ±16 bpm at rest in dry conditions, with performance significantly decreasing for activity in water (accuracy: −57 bpm; precision: ±68 bpm). However, it should be noted that correlation strength is low for tests performed in-water during activities; thus, in the future, it would be interesting to carry out studies aiming at optimizing the measurement procedure for applications of PPG-based wearable devices in swimming activities.

It is extremely important to validate a wearable device in the specific operating conditions where it will be employed, since its performance can be affected by specific boundary conditions, and specific influencing inputs can be present. In swimming applications, water undoubtedly plays a relevant role in the determination of the signal quality and, consequently, on the metrological performance of the devices. Wearable devices can undoubtedly represent a powerful tool for supporting athletes and coaches during training and help to assess performance in competitions. However, their metrological performance should always be appropriately considered, to be able to adequately interpret the results and infer reliable considerations, which can effectively provide feedback to both athletes and coaches. In this context, the definition of the test protocol for sensors validation is fundamental. It is clear that the protocol proposed by the authors can be adapted to different contexts, not limited to sports applications or the analysis of the cardiac rhythm, but considering, for example, the athlete’s activity performed during a training session. The validation protocol needs to mirror the real operating conditions to consider all the interfering factors in the evaluation and provide metrological results specific for the exact context. On the other hand, it would be of interest to develop a wearable device specific to in-water applications, considering the results from the validation procedure and exploiting them to optimize the measurement chain, starting from the skin-sensor interface to processing techniques aiming at enhancing data quality.

In the future, it would be interesting to widen the test population to include a great amount of physiological variability (in terms of age, skin tone, gender, and so on), whose effects would reflect on the results.

## Figures and Tables

**Table 1:** Main characteristics of the tested population.

Subject No.	Age [Years]	Weight [kg]	Height [m]	BMI [kg/m^2^]
1	16	63	1.68	22.32
2	14	48	1.60	18.75
3	18	47	1.56	19.31
4	18	55	1.65	20.20
5	18	47	1.62	17.91
6	13	48	1.60	18.75
7	13	53	1.60	20.70
8	19	52	1.58	20.83
9	22	53	1.65	19.47
10	22	55	1.67	19.72

**Table 2:** Technical specifications of the wearable devices employed in the study.

Wearable Device	Measured Parameters	Sensing Technology	HR Measurement Technical Specifications
Polar H10	HR, RR	ECG electrodes	Sampling frequency (ECG): 130 Hz
Polar Vantage V2	HR, activity, sleep, steps, distance, energy expenditure, velocity	PPG	Sampling frequency (RR series): 1 Hz Measurement range: 15–240 bpm
Garmin Venu Sq	HR, respiratory rate, blood oxygen saturation (SpO_2_), sleep, steps, distance, energy expenditure, activity, VO_2max_	PPG	Sampling frequency (RR series): 1 Hz

**Table 3:** Metrological performance of the tested smartwatches in terms of measurement accuracy (µ) and precision (±2σ), confidence interval at 95% (CI95%), mean absolute percentage error (MAPE), and Pearson’s correlation coefficient (ρ)—dry vs. in-water conditions.

Testing Conditions	Tested Smartwatch	µ [bpm]	±2σ [bpm]	CI 95% [bpm]	MAPE [%]	ρ [-]
Dry conditions	Garmin Venu Sq	1	13	[−14, 12]	4.05	0.95
	Polar Vantage V2	−5	23	[−27, 18]	8.00	0.85
In-water tests	Garmin Venu Sq	−44	74	[−117, 30]	29.95	0.26
	Polar Vantage V2	−14	60	[−74, 46]	17.17	0.59

**Table 4:** Metrological performance of the tested smartwatches in terms of measurement accuracy (µ) and precision (±2σ), confidence interval at 95% (CI95%), mean absolute percentage error (MAPE), and Pearson’s correlation coefficient (ρ)—dry vs. in-water conditions, separating tests at rest and during activities.

Testing Conditions		Tested Smartwatch	µ [bpm]	±2σ [bpm]	CI 95% [bpm]	MAPE [%]	ρ [-]
Dry conditions	At rest	Garmin Venu Sq	−1	16	[−17, 15]	4.83	0.65
		Polar Vantage V2	−5	19	[−24, 13]	7.32	0.32
	During activity	Garmin Venu Sq	−1	12	[−13, 11]	3.60	0.95
		Polar Vantage V2	−4	24	[−28, 19]	8.29	0.83
In-water tests	At rest	Garmin Venu Sq	−12	41	[−52, 28]	17.32	0.32
		Polar Vantage V2	−4	28	[−32, 24]	10.37	0.62
	During activity	Garmin Venu Sq	−57	68	[−124, 10]	58.94	0.13
		Polar Vantage V2	−18	68	[−84, 49]	29.78	0.2

**Table 5:** Metrological performance of the tested smartwatches in terms of measurement accuracy (µ) and precision (±2σ), confidence interval at 95% (CI95%), mean absolute percentage error (MAPE), and Pearson’s correlation coefficient (ρ)–results obtained at rest vs. during activities.

Test Conditions	Tested Smartwatch	µ [bpm]	±2σ [bpm]	CI 95% [bpm]	MAPE [%]	ρ [-]
At rest	Garmin Venu Sq	−6	31	[−37, 25]	10.17	0.42
	Polar Vantage V2	−5	24	[−28, 19]	9.36	0.67
During activity	Garmin Venu Sq	−23	70	[−93, 47]	16.15	0.20
	Polar Vantage V2	−10	48	[−58, 39]	12.59	0.69

## Data Availability

The data presented in this study are available on request from the corresponding author.
